# Letter from Dr. J. L. Levison

**Published:** 1858-10

**Authors:** J. L. Levison

**Affiliations:** 7 Kentridge Villa, St. John's Wood, London


*Letter from Dr. J. L. Levison.*—The following letter was received too late for publication in the proper place in the present number of the Journal,—we, therefore, insert it in the editorial department. The teeth referred to in the letter, which were extracted by Dr. L., were sent with it to the Editors, and will be placed in the Museum of the Baltimore College of Dental Surgery.—Eds.

### *Two Teeth in the Mouth at Birth.*

To the Editors of the Quarterly Review of Dental Surgery.

GENTLEMEN,—When in practice in Hull, in the years 1825 and 1826, I made many friendships among the medical men of that town, and was always consulted by them in any novel or *outré* cases of Dental Surgery.

Among these patrons there was one Mr. C. with whom I was on terms of the greatest intimacy and confidential companionship, from a kindred love of science. One day my friend told me that his sister had given birth to a fine female child, who had two teeth (central incisors) of the upper jaw when she was born, and asked what should be done?

Why they should be extracted, as they are merely extraneous bodies, and will induce great local irritation.

"I think your advice judicious, but when should they be extracted?"

Why immediately—for so true it is in incipient disease,

> "That if a thing should be done, t'were well done,
> If it were done quickly."

It may be remarked, incidentally, that he read of the crooked-backed Richard III, who counted among other phenomena which marked him for a malignant course,—"That he was born with teeth in his head!" And this could not have been the cause, *per se*, as there was nothing malignant in the little lady we are speaking of, or any extraordinary signs or indications to give her any peculiarity.

She was pretty, very thin and very fretful. The called "infirmity of temper," I pronounced to be the irritation of the teeth as foreign bodies. The mother, however, resisted for a time all arguments or advice for the removal of the teeth, and only ultimately yielded when the life of the child seemed jeopardized. So one morning, having been specially summoned, I performed "this minor operation" on the youngest patient I ever had to extract teeth from, and as she did not know any thing about it, so trifling was the whole affair, that she scarcely simpered.

But very soon, probably in a fortnight, the results of the operation were well marked. The little maiden grew stout and strong, and her once pale face become tinged with "roseate hues," and her soft pasty flesh acquired more of the tone of muscular fibres. She crowed merrily, and her features were suffused with smiles whenever noticed, and all symptoms of fretfulness ceased.

I lived to see her a married woman and know that she had a family, but never heard that any of her children had any similar development of dental organs. So we must attribute this novelty to a freak of nature, or to the local energy in the *vis formativa* in connection with the two pretty little teeth I send you—whilst all the others came in their "season" in a more normal manner.

I am, gentlemen, yours most respectfully,

J. L. LEVISON.

7 Kentridge Villa, St. John's Wood,
*London, September 14th,* 1858.

